# Anatomical-Based Imaging of Cystic Echinococcosis and Review of the Current Literature

**DOI:** 10.5152/eurasianjmed.2022.22309

**Published:** 2022-12-01

**Authors:** Mustafa Yesilyurt, Veysel Esdur

**Affiliations:** 1 Radiology Clinic, Regional Training and Research Hospital, Erzurum, Turkey

**Keywords:** Cystic echinococcosis, hydatidosis, clinic administration, diagnosis

## Abstract

Echinococcosis is a zoonosis caused by cestodes of the genus *Echinococcus*. This serious disease continues to be an important public health problem as it is endemic in many parts of the world. Cystic disease affects many organs in the body, most commonly the liver. Hydatid disease can cause a diverse spectrum of symptoms, from asymptomatic infection to potential death. In the presence of clinical suspicion, the diagnosis is made by serology and imaging methods. Imaging findings can range from completely cystic lesions to completely solid appearance and calcification. The imaging method to be used depends on the involved organ and the stage of the cyst. The updated recommendations of the World Health Organization-Echinococcosis Informal Working Group for the stage and treatment of human echinococcosis have had important implications. Accordingly, there are 4 approaches to the clinical management of hydatid disease: surgery, percutaneous techniques and drug therapy for active cysts, and a “watch and wait” approach for inactive cysts. Since it directly affects the treatment, it is necessary to be familiar with the imaging findings of the cyst, especially in endemic areas.

**Main Points**

- Echinococcosis is a serious public health problem that is still endemic in certain parts of the world. Imaging methods, especially ultrasound, are very important in diagnosis. The World Health Organization classification for cystic echinococcosis (CE) in ultrasound is a powerful tool that clinicians should be familiar with to guide treatment.
- In cases with CE, a multidisciplinary treatment approach should be followed to prevent complications and overtreatment. Cysts in the active phase should be evaluated in terms of interventional and/or medical treatment, and a wait-and-see approach should be applied in inactive cysts.
- Cystic echinococcosis primarily affects the liver and typically shows well-known, characteristic imaging findings. However, there are many possible local complications. It can be seen in almost any anatomical location secondary to hematogenous spread and rupture.
- Cystic echinococcosis should be considered in the differential diagnosis of cystic lesions in any anatomical localization, especially in endemic areas.

## Introduction

The term echinococcosis refers to two serious zoonotic diseases, cystic echinococcosis (CE) and alveolar echinococcosis (AE), caused by *Echinococcus granulosus* and *Echinococcus multilocularis*, respectively.^1-3^ *Echinococcus granulosus* is the more common type, while *Echinococcus* *multilocularis* is rarer but more invasive and mimics a malignancy.^2-5^ Hydatid cyst consists of the larval form of *Echinococcus granulosus* and is the most common tumor in the liver parenchyma due to parasite infestation.^6^

The annual incidence of CE is between 1 and 200 per 100,000 in endemic areas.^7-9^ It is endemic in many countries in South America, the Middle and Far East, and around the Mediterranean.^10^ The annual incidence in Turkey is 50 cases per 100,000 people.^11^ Echinococcosis has been listed as 1 of 17 neglected diseases by the World Health Organization (WHO) and is aimed to be controlled or eliminated by 2050.

Involvement sites can be listed as liver (75%), then lung (15%), and then other organs (10%).^12^ Cystic echinococcosis can cause many potential local complications such as exophytic growth and compression on surrounding organs, portal vein involvement, biliary communication, perforation, peritoneal insemination, and transdiaphragmatic thoracic involvement.^3,10^

In this article, parasitology, various organ involvement, and radiological findings of hydatid disease were discussed. Cases from our experienced department, which encounter approximately 500 cases of hydatid cysts per year, will be presented.

## Parasitology

Hydatid cyst disease occurs in humans as a result of contamination with *Echinococcus granulosus* larvae. Generally, carnivores such as dogs are definitive hosts and sheep are intermediate hosts, whereas humans are accidental hosts. The disease is caused by ingesting food or water contaminated with infected dog feces. Ingested larvae invade the intestinal mucosa, enter the portal circulation, and develop into a cyst in the liver. In the literature, there are studies stating that the cysts in the liver grow 1 cm in the first 6 months and then approximately 2-3 cm per year depending on the host tissue resistance.^3,4^

## Clinical Features

The clinical features of CE are usually related to the relevant organ and are non-specific. Cysts may present different clinical features depending on the organ they are located in. Approximately 60%-75% of CE cases do not cause clinical signs, especially in the early stages of infection, and are incidental.^13^ The vast majority of CE cases go undetected in childhood. Cystic echinococcosis cysts mostly affect the liver (70%), the lungs (20%-30%), or both, although less frequently they can occur in other organs.^10,14-16^ Symptoms result from compression or displacement of surrounding tissue, depending on the location and size of the cysts.^16-18^

In the literature, it has been reported that the incidence of complications in hydatid cyst cases is as high as 60%. The most important complications are the involvement of neighboring structures and fistula development, and the spread of juvenile cysts to the biliary tract, peritoneal cavity, pleura, and bronchi as a result of the rupture of the cyst.^14,19,20^ Cholangitis and/or cholestasis resulting from cystobiliary fistula may be the reason for admission to the clinic. The mortality rate in CE cases is reported to be approximately 2%-4%.^9,21,22^

## Diagnosis

The diagnosis of CE is based on clinical findings, imaging techniques and serology, depending on the location of the cyst. Microscopic examination of the fluid can reveal the presence of protoxes.^23^ In some uncertain cases, serology may be useful to confirm the diagnosis, but the sensitivity of serology is variable. False-negative results may occur in immature, inactive, or extrahepatic cysts. Positive serology is not associated with viability and may persist for years even after curative surgery. Therefore, it is not suitable for follow-up.^22,24,25^ The standard diagnostic method for hepatic CE is ultrasound (US).^23^

## Imaging

Imaging is the gold standard in the diagnosis of CE.^26^ Imaging has also been effective in recognizing the transformation of the cyst from living to dead cysts, staging the disease, understanding its relationship with the surrounding anatomical structures, and recognizing complications such as secondary bacterial abscess formation, cystobiliary and cystobronchial fistula.

Ultrasonography plays a fundamental role in the imaging of abdominal localized cysts. If peripherally located, cysts in other organs, including the lung, can be visualized by US.^27^ Cyst staging is based on US characteristics (Gharbi and WHO classifications). Today, 4 different approaches to the treatment of CE are presented based on the WHO cyst staging.^28^

Other imaging modalities used secondarily for hepatic CE lesions are magnetic resonance imaging (MRI) and computed tomography (CT). While cysts’ features can be well described by MRI, they cannot be clearly demonstrated by CT.^29,30^ These methods are generally used for preoperative evaluation or in case of complications.

The imaging modalities to be used in diagnosing hydatid cyst in pregnancy are US and MRI.^31^

If US is difficult to diagnose in the presence of patient-related difficulties and complications, CT indication arises. Intravenous contrast agent administration is required in the presence of suspected complications.^32^

Features specific to CE can be easily diagnosed by MRI as in US. In addition, the internal architecture of dead cysts that cannot be evaluated due to artifact due to calcification can be clearly visualized by MRI. The peripheral hypointense rim, which is a clue for CE, can sometimes be seen on T2-weighted images and aids in the diagnosis.^3^ It may be difficult to diagnose without typical MRI findings, especially in inactive CEs. In addition, recent studies have shown that diffusion-weighted images play an active role in diagnosing tumoral and infectious lesions of internal organs. In addition, hepatobiliary-specific agent-enhanced MR cholangiography demonstrates the anatomical localization of bile leakage. In addition, it provides direct visualization of contrast agent extravasation to fluid collections. Thus, it allows the detection of biliary complications of hydatid cysts.^10^

## World Health Organization Classification of Cystic Echinococcosis

In 1985, WHO established the Informal Working Groups working in the field of Echinococcosis. These groups were merged into a single group by WHO in 1995 under the name of the Echinococcosis Informal Working Group (WHO-IWGE). The mission of this group is to strengthen the prevention and control of echinococcosis through effective cooperation with relevant sectors.

The WHO-IWGE introduced a standard classification of CE in 1995. In 2009, the WHO-IWGE published consensus and updated guidelines for the diagnosis, treatment, and management of CE and AE. The final version of the pathognomonic features of CE cysts in US, as updated by the WHO, is listed in Table 1.^13,33,34^ This classification can distinguish between active, transitional, and inactive cysts—based on the size, number, morphology, and localization of the cyst(s). Thus, it contributes to further management and treatment.

In the international classification, CE type 1 (CE1) and CE type 2 (CE2) correspond to “active stages,” CE type 3 (CE3) a and b to “transition stage,” and CE type 4 (CE4) and CE type 5 (CE5) to “degenerate stages.”^35^

**Figure 1. A-F.**
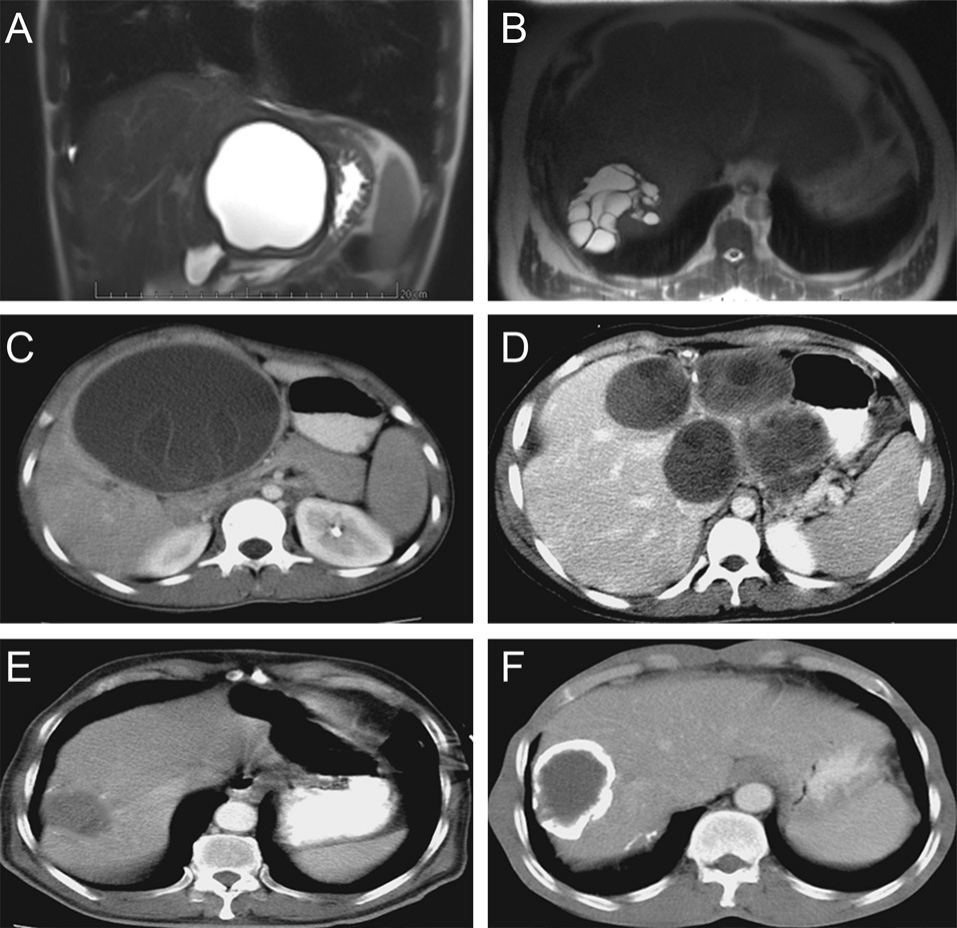
Demonstration of differential diagnosis of cystic echinococcosis by computed tomography (CT) and magnetic resonance imaging (MRI) according to the cyst stage. (A) Hyperintense hepatic cyst with double-line sign considered pathognomonic for World Health Organization (WHO) CE1 cysts, T2-weighted MRI. (B) Multivesicular cystic echinococcosis hepatic cyst WHO CE2 cyst, T2-weighted MRI. (C) Hypodens hepatic cyst with detached endocyst (water-lily sign) WHO CE3A cyst, CT. (D) Daughter cysts inside a solid cyst matrix WHO CE3B cyst, CT. (E) Heterogeneous, parasitic membranes embedded in avascular solid contents WHO CE4 cyst, CT. (F) Cysts with degenerative content and heavily calcified wall WHO CE5 cyst, CT.

CE1: Cystic echinococcosis type 1 cysts are double-walled uniloculated cysts (Figure 1A). These cysts are fertile cysts containing viable protoscolices.^35^ Depending on the size of the cyst, medical, surgical, and puncture, aspiration, instillation, and reaspiration (PAIR) treatments can be applied.

CE2: Cystic echinococcosis type 2 cyst is a uniloculed cyst with avascular septum resembling daughter cysts^35^ (Figure 1B). Daughter cysts and detached endocysts (“lotus sign”) are often overlooked on CTs but are clearly visible on US and MRI. Except for PAIR, other treatment methods can be applied according to the size of the cyst. The PAIR is contraindicated for CE2.^23^

CE3A: Cystic echinococcosis type 3A cyst consists of thin and regular membranes seen inside the cyst with the decomposition of the endocyst (Figure 1C). The subclassification of CE3 as CE3a and CE3b supports a recent study using high-field MR spectroscopy to evaluate the ex vivo metabolic profile of cyst content.^29,35^ According to this study, CE3a may be alive or dead, whereas CE3b is constantly alive. It should be noted that CE3a and CE3b also respond differently to nonsurgical treatments.^36^ As in CE1, all treatments can be applied depending on the size of the cyst.

CE3B: Cystic echinococcosis type 3B cyst is a cystic lesion with solid component predominantly solid with daughter cysts (Figure 1D). Daughter cysts within a solid cyst matrix are usually not recognized by CT. As with CE2 cyst, PAIR is contraindicated.^23^

Ultrasound is the most sensitive method for visualizing the double wall sign, hydatid sand, and septa in CE.3 For CE1, CE2 and CE3, “snowflake mark,” “thick cyst wall,” “lotus mark,” and “wheel-like” specific images are the main features revealed by US.37

CE4: The typical appearance of a CE4 cyst is heterogeneous, parasitic membranes embedded in avascular solid contents (Figure 1E). These cysts often cannot be diagnosed with CT because of their unique canalicular structure. Therefore, CE4 cysts can be mistakenly interpreted as CE1 cysts.

CE5: These cysts are in the form of cysts with solid contents with eggshell wall calcifications (Figure 1F). Cystic echinococcosis type 4 and CE5 are inactive cysts that have lost their fertility and degenerate. With the latest development in WHO classification, it has been revealed that calcifications are not limited to CE5 cysts, they can be present in all cystic stages to varying degrees and therefore do not indicate cyst death.^30^

The density of CE1, CE2 and CE3A cysts is generally similar to water (3-30 Hounsfield units). The rare “wool ball” appearance may aid in the diagnosis of type CE4. It cannot clearly distinguish solid and semi-solid type CE4s from abscess and tumor.

Wall or matrix calcifications are significant for the CE5 type, whereas posterior acoustic shadowing prevents the evaluation of the lesion’s matrix.^35,38,39^ Therefore, the diagnosis of CE4 and CE5 requires more advanced imaging modalities such as CT and MRI. All of the components of the CE5 type cyst may show dense calcification that can be easily detected on CT.^40^ The imaging method that best reveals calcification in cysts is CT. In the literature, there are studies recommending regular monitoring of symptomatic and uncomplicated inactive CE4-CE5 cysts with imaging techniques with a so-called “watch and wait” approach.^26^

## Involvement of Various Organs

**Figure 2. A, B.**
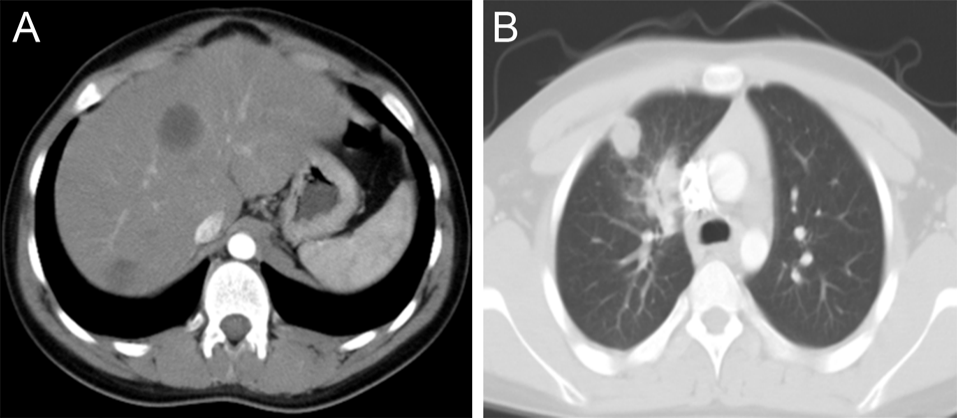
Computed tomography scans of the same patient show hydatid cyst in the right lung upper lobe anterior segment (A) and liver right lobe (B).

The most common site of involvement in the study of Polat et al^4^ liver (74.8% of cases). Patients with hepatic involvement had isolated hepatic involvement in 48.3%, liver and lung involvement in 26.9% (Figure 2), and combined involvement of organs other than the liver and lung in 24.7%.

The second most common site of involvement was the lung with 24%. Of these, 83.1% showed isolated lung involvement. Other sites of involvement (in decreasing order of frequency) include the peritoneum, spleen, kidney, brain, mediastinum, heart, bone, soft tissues, spinal cord, pleura, adrenal glands, bladder, ovary, scrotum, and thyroid gland.^4^

### Liver

**Figure 3.**
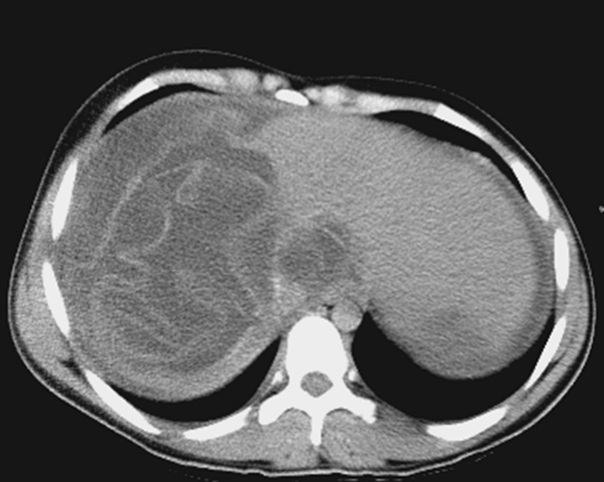
Non-contrast abdominal computed tomography scan shows rupture of the right lobe hydatid cyst of the liver into the perihepatic area.

**Figure 4. A,B.**
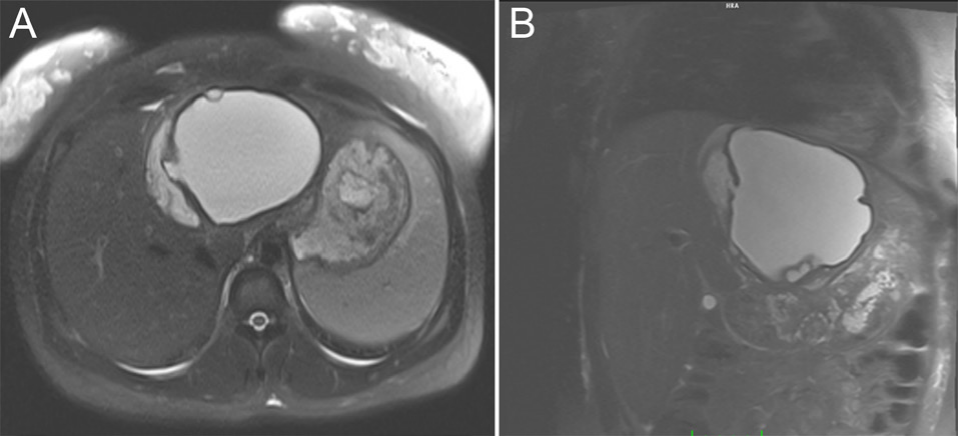
Axial (A) and coronal (B) T2-weighted magnetic resonance image shows intrahepatic rupture of the left lobe hydatid cyst of the liver.

Clinically, most CE patients present to clinics or hospitals late. Population screening has shown that liver CEs in humans grow very slowly, with most cysts showing no change in size within 10 years and a third growing less than 3 cm.^41^ The mean cyst growth was 0.7 cm in cases followed for a long time.^41,42^ Clinical symptoms in cases of CE usually occur when a cyst in the liver is more than 10 cm in diameter or when more than 70% of the organ volume is occupied by a cyst or cysts.^43^ Patients with CE with symptomatic liver cysts most often present with anorexia and upper abdominal pain. Compression of the biliary tract can cause jaundice. Palpation may reveal distension, a tumor-like mass, and hepatomegaly. Rupture of the cyst may occur spontaneously with increased intracystic pressure or secondary to surgery or trauma (Figures 3 and 4). Rupture has been reported to occur in approximately 3%-3.2% of patients with liver hydatid cysts.^20,44^ Other complications are infection of the cyst, fever, urticaria, eosinophilia, and less commonly anaphylaxis.^45^ Diaphragm and thoracic cavity involvement is seen in 0.6%-16% of hepatic CE cases.^46^ Intrathoracic rupture of CEs located in the hepatic dome is a serious complication resulting in damage to the pleura, pulmonary parenchyma, and bronchi.

### Lung

**Figure 5. A-C.**
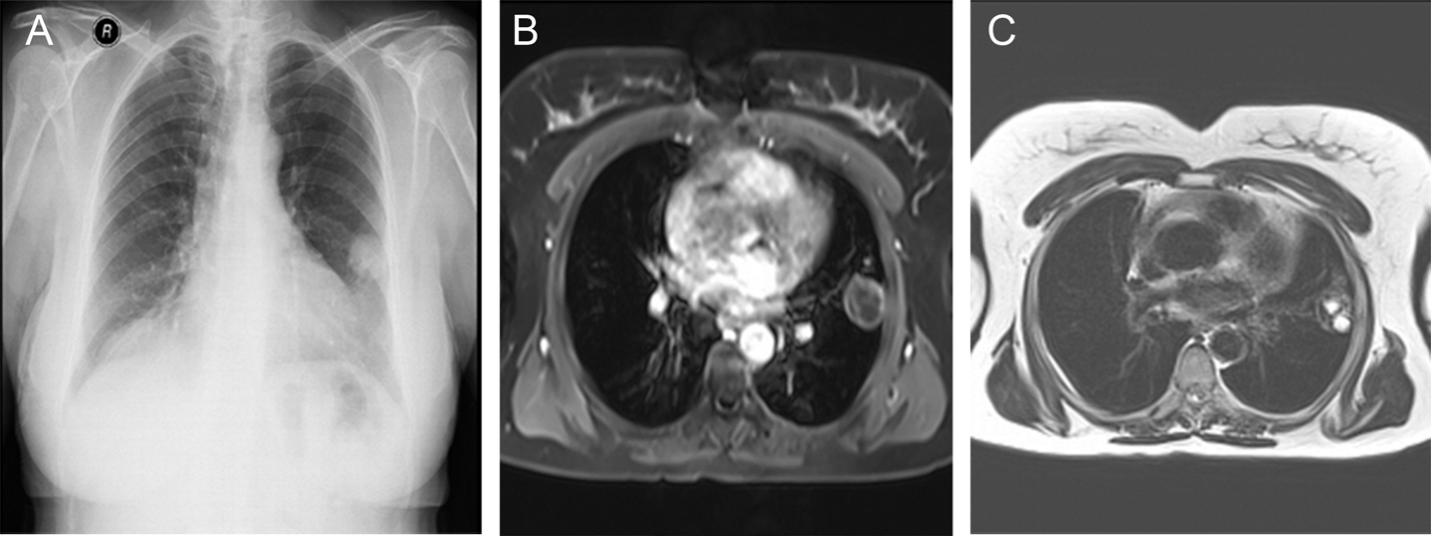
Posteroanterior chest x-ray (A), fat-suppressed contrast-enhanced T1-weighted magnetic resonance image (MRI) (B), and T2-weighted MRI (C) showing the left pulmonary hydatid cyst.

**Figure 6.**
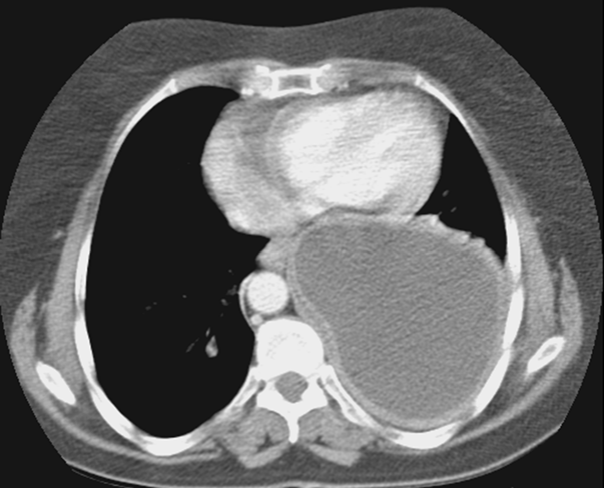
Contrast-enhanced computed tomography scan of thorax showing giant hydatid cyst of left lung.

**Figure 7. A-C.**
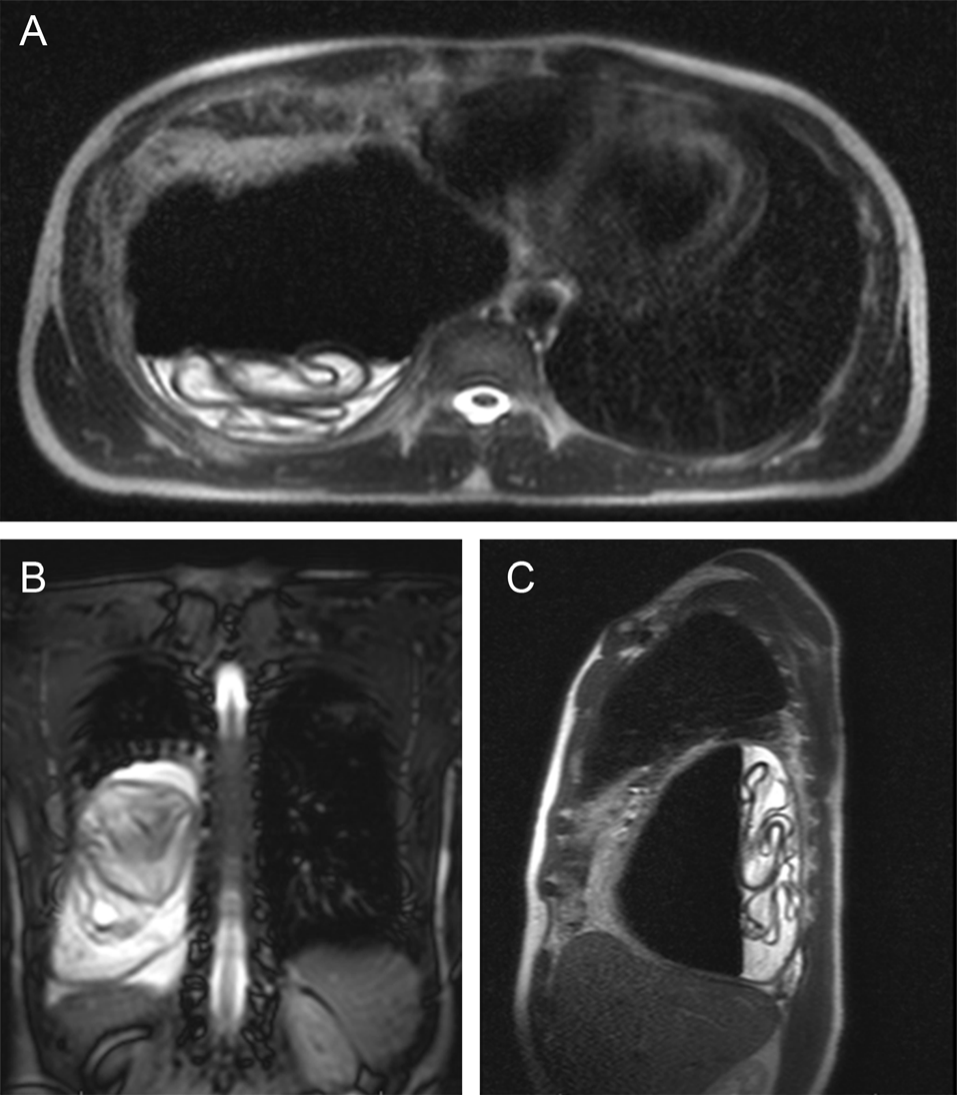
Axial (A), coronal (B), and sagittal (C) T2-weighted magnetic resonance image shows rupture of giant hydatid cyst in the right lung.

The lung is the second most frequently involved organ after the liver. Due to the slow growth of the cyst, the disease can occur in adulthood, although acquired early. Pulmonary CEs prefer the right posterior lung segments and 60% of cases occur in the lower lobes (Figure 5). Bilateral involvement is seen in 20% of cases and multiple cysts in 30%. Simultaneous involvement of the liver and lungs is seen in approximately 6% of all patients with CE of the chest and abdomen. Pulmonary CEs can range from 1 to 20 cm in diameter (Figure 6). Because of their compressibility, the lungs are the only organ in which CEs can grow this large.^4^ Compression symptoms and complications resulting from the growth of the cyst are the cause of the presentation in extrahepatic involvement. Cysts larger than 5 cm usually cause bronchial compression. Common complications of pulmonary hydatidosis include cyst rupture, suppuration, and secondary infection (Figure 7). Symptoms secondary to the cyst include sudden onset of chest pain, cough, and fever.

The diagnosis is made by CT supplemented with serology and chest x-ray. It may be accompanied by leukocytosis, eosinophilia, and a high erythrocyte sedimentation rate. Computed tomography characteristics of CEs are smooth walls of varying thickness and homogeneous internal water content or near-water density. The pericyst, lamina, and germinal layers of the cyst adhere to each other and therefore appear as a single wall.^47^ If air enters the space between the pericyst and ectocyst, separation of the parasitic membranes from the pericyst occurs. This is called the “breaking sign.” This feature is known as the “crescent” or “meniscus sign,” which is a reliable but non-pathognomonic marker for pulmonary hydatid disease.^4^

Regardless of the size of the pulmonary cyst and whether it is intact or ruptured, the preferred treatment method is surgery.^48^

### Peritoneum-Retroperitoneum

**Figure 8.**
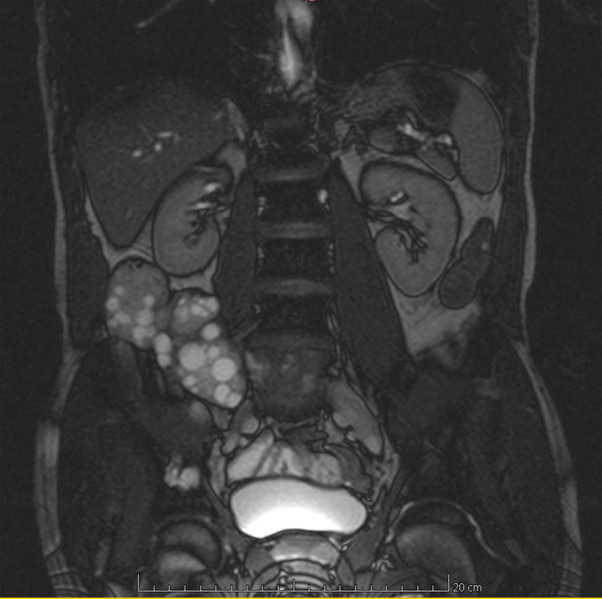
Demonstration of a retroperitoneal multilocular hypointense cystic lesion extending from the right ileopsoas muscle to the subhepatic area on coronal T2-weighted abdominal magnetic resonance image.

Peritoneal-retroperitoneal CE is usually secondary to rupture or surgical grafting of a hepatic cyst. Primary involvement is extremely rare (Figure 8).^49^ Most CEs are symptomatic, and their symptoms are variable and never pathognomonic. Symptoms depend on the organs involved, their localization in the affected organ, the size of the cysts, and the interaction between the cyst and adjacent organ structures.^49^ Imaging findings were similar to those of other organ CEs. All types of CE can be seen in the retroperitoneum.^4^

It has been reported in the literature that peritoneal cysts develop in approximately 5%-14% of patients with liver hydatid cysts.^50,51^ The number and size of peritoneal hydatid cysts are variable. Unusual complications such as pelvic venous congestion may also occur as a result of compression secondary to a giant peritoneal hydatid cyst.^52^

### Spleen

**Figure 9.**
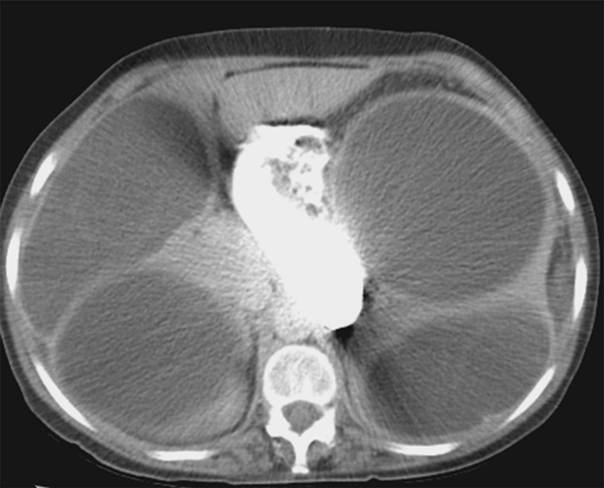
Computed tomography showing hydatid cysts of the liver and spleen.

Primary spleen involvement in CE (2.5%-5.8%) ranks third after liver and lung in terms of frequency.^53^ Although isolated spleen involvement is very rare, it may accompany other organ involvements, often liver (Figure 9). It usually develops via the systemic route or intraperitoneal spread secondary to cyst rupture. Splenomegaly, upper quadrant abdominal pain, and fever are the most common clinical manifestations.^54^ The imaging features of splenic hydatid cysts are generally similar to hepatic hydatid cysts and are solitary. Computed tomography can show the typical high attenuation linear wall and calcification occurring in the cyst.

### Kidneys

Renal involvement is seen in approximately 2%-4% of cases.^55,56^ Renal function is usually preserved in cases of renal hydatid cysts.^57^ Depending on its localization in the kidney, it may remain asymptomatic for years. The most common symptoms are dysuria, flank pain, and a feeling of fullness in the flanks. Rupture of the cyst into the collecting system develops in approximately 18% of cases.^58^ As a result, cyst hydraturia and acute renal colic occur. Ring calcification seen in the cyst wall on CT suggests hydatid cyst. Renal hydatid cysts can be confused with cystic nephroma, simple cysts, and renal cell carcinoma. In most cases, the diagnosis is made by percutaneous puncture and serology.^59^

## Unusual Locations

### Brain

**Figure 10. A,B.**
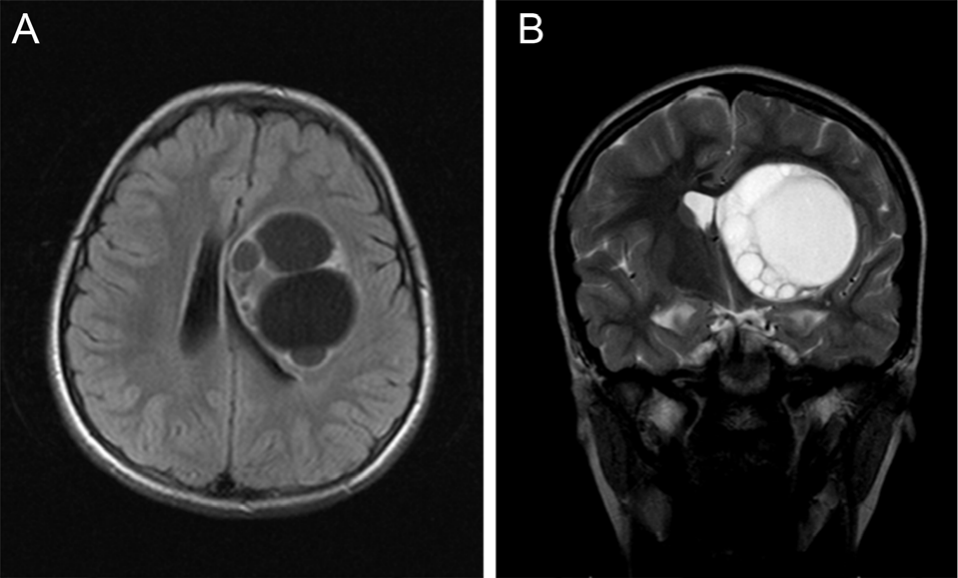
Axial FLAIR (A) and coronal T2-weighted (B) magnetic resonance image of the brain demonstrating a well-defined T2 hyperintense lesion that is totally suppressed on the FLAIR sequence, with no sign of perilesional edema.

Cerebral localization is very rare and constitutes approximately 1% of hydatid cyst cases.^55,60^ Even in endemic areas, approximately 2% of intracranial masses are hydatid cysts.^4^ Cerebral CE is more common in childhood than in adults. Cerebral CE is usually seen in the middle cerebral fossa in the supratentorial area (Figure 10). In children or young patients, a cyst is suspected by bone erosion or suture separation on head radiographs.^12^ On CT and MRI, a mass lesion with attenuation or intensity similar to the cerebrospinal fluid is seen. Calcification is extremely rare. After intravenous contrast agent injection, no enhancement is seen in the lesion.^12^

Cerebral CE does not cause edema in the surrounding brain parenchyma but creates a mass effect; this feature is important in differentiating it from abscess and cystic masses (Figure 10).^4^ One of the characteristic features of cerebral CE is the presence of a hypointense rim on T2-weighted MR images.^4^ Cerebral CE is solitary, except for spontaneous rupture or secondary to trauma. Cerebral CE is usually solitary. It can be multiple if the cyst ruptures spontaneously or secondary to trauma.

### Bone

Bone involvement in CE is approximately 0.5%-4% of cases.^59,61,62^ The sites of involvement are the spine, pelvis, femur, tibia, humerus, and skull bones, in order of frequency.^4,62^ Pericyst does not form in the bone, so aggressive growth is observed along channels with less resistance.^61,63^ After the cysts reach the cortex and destroy it, it spreads to the surrounding tissues. As a result of the rigidity of the bone structure, the cyst cannot take its typical shape. Calcification is very rare in intraosseous cysts.^4^

**Figure 11. A-C.**
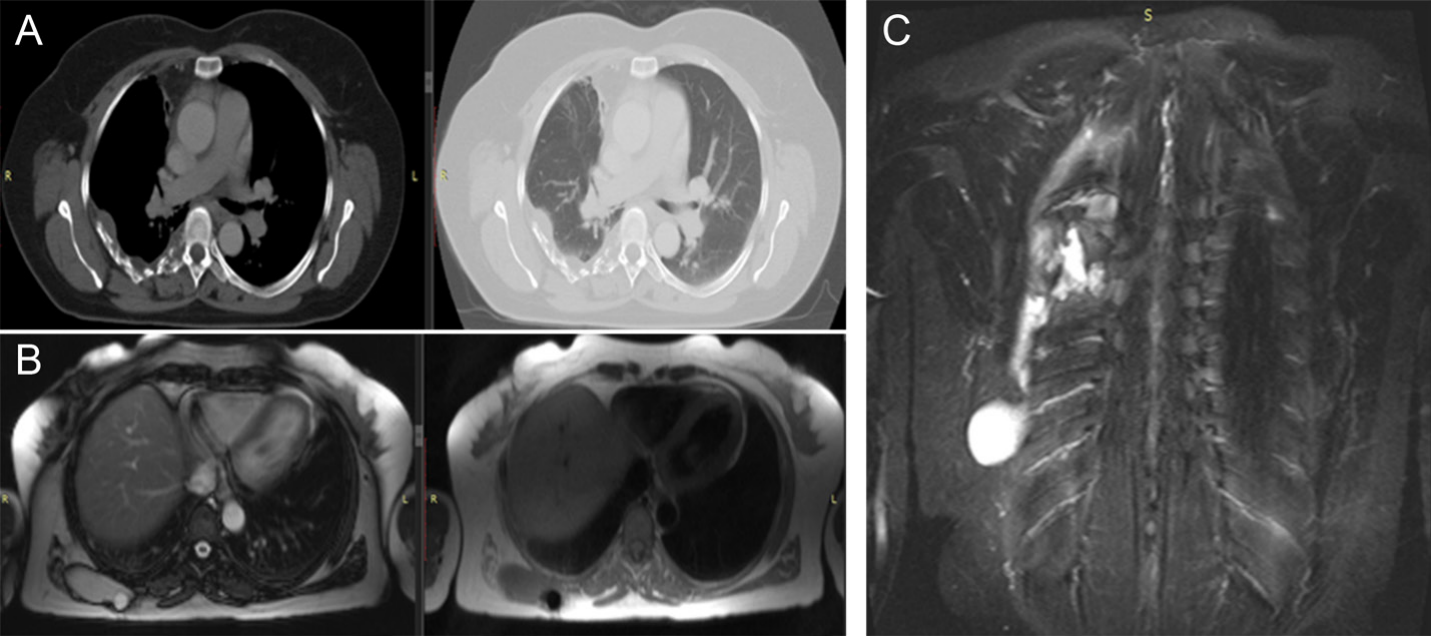
Thoracic computed tomography (A) scan showing intramedullary lytic and enlarging masses posterior to the fourth, fifth, and sixth ribs on the right. A hydatid cyst is seen in the right latissumus dorsi muscle on the T1- and T2-weighted thorax magnetic resonance image (MRI) (B) of the same patient. Demonstration of cysts in the same patient on coronal T2-weighted thorax MRI (C).

In bone CE, the cyst is usually multiloculated, well-circumscribed, and osteolytic. There may be cortical thinning, bony enlargement, and spread to adjacent soft tissues (Figure 11). Sclerosis and periosteal reaction are not expected.^59^

### Pancreas

**Figure 12.**
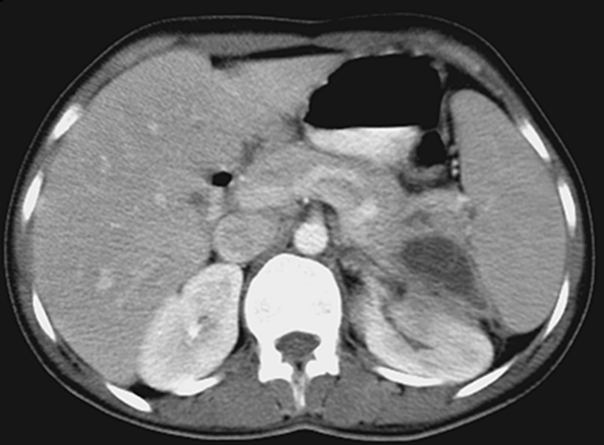
Contrast-enhanced computed tomography scan of the abdomen showing cystic lesions with a curvilinear laminated membrane at the tail of the pancreas.

The pancreas is one of the rarely involved organs and its incidence in various regions varies between 0.19% and 2% (Figure 12).^2,4^ The hydatid cyst reaches the pancreas in a hematological way and rarely by retroperitoneal spread.^64^ Clinical symptoms depend on the localization of the cyst in the pancreas, its size, and the involvement of the biliopancreatic system.^2^ The main symptoms are abdominal pain and vomiting. Obstructive jaundice, recurrent acute pancreatitis, weight loss, and/or epigastric mass are common causes of hospital admission.^65^ Ultrasonography shows the thick wall of the cyst and daughter vesicles. CT, on the other hand, can help the diagnosis by showing the cyst with annular calcification.^65^ Definitive diagnosis is usually made by surgery.

### Adrenal Gland

Adrenal gland involvement of hydatid cyst is very rare, but it has been reported as 0.5% in the literature.^66^ Parasitic cysts involving the adrenals are usually secondary to generalized echinococcosis. Rarely, primary adrenal gland involvement is seen. Adrenal cysts are often asymptomatic. The most common symptoms are blunt flank pain, gastrointestinal symptoms, and a palpable mass.^67^ Because adrenal cysts are mostly asymptomatic, they are usually incidentally found in imaging studies.^68^ Acute abdominal pain may develop as a result of bleeding, rupture, or infection into the cyst. Anaphylactic shock may develop as a result of rupture of the hydatid cyst.^68^

**Figure 13. A,B.**
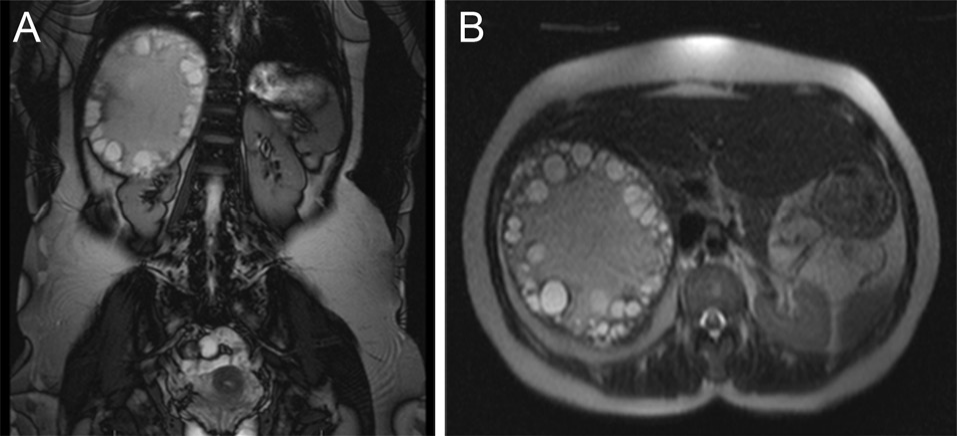
Coronal (A) and axial (B) T2-weighted magnetic resonance image of the abdomen demonstrating right adrenal cystic mass with daughter cysts inside a mucinous or solid cyst matrix.

The diagnosis of adrenal hydatid cyst is made by revealing cystic lesions, daughter vesicles, and calcifications by US, CT, and MRI (Figure 13).

### Spinal Cord

Spinal cord CE is a rare form that accounts for less than 1% of all cases.^4^ According to the decreasing frequency of involvement, the sites of involvement are thoracic, lumbar, sacral, and cervical spine. Spinal cord CE is divided into 5 types: vertebral, paravertebral, intramedullary, extradural intraspinal, and intradural extramedullary. The first 2 types are the most frequent.^4,69^ They are usually seen as multiple cysts. No contrast enhancement is observed after intravenous contrast agent injection. Calcification is very rare in spinal cord CE.^4^

### Soft Tissue

Soft tissue CE is seen in endemic areas in approximately 2.3% of cases.^59^ It is often associated with the involvement of other organs such as bone (Figure 11). Ultrasound, CT, and MRI are helpful in locating the cyst, but imaging findings are not specific. The growth of the cyst in the muscle is difficult due to the presence of lactic acid and contractility. Therefore, the affinity for the muscles is high in the neck, trunk muscles, and roots of the limbs. The increased vascularity of the muscles in this region also has a great effect on this. Cases of primarily muscle involvement are very few in the literature.^70,71^ In the case of spontaneous rupture, trauma or surgical rupture, more than 1 intramuscular cyst may be seen.

### Heart-Pericardium

Cardiac hydatid cysts account for 0.5%-2% of all cases in endemic areas.^72-74^ Involvement can usually be hematogenous or due to rupture of lung CE. The cyst is found in the ventricles and more rarely in the pericardium. Transthoracic echocardiography, CT and MRI are used for diagnosis and to show the nature of the cystic mass and its relationship to the heart chambers and adjacent structures. Transthoracic echocardiography may be insufficient to define the relationship of the cyst with neighboring structures. Computed tomography and MRI are ideal for more accurate evaluation of all aspects of cysts. An important limitation of CT in the evaluation of cardiac and pericardial CE is an artifact due to cardiac motion. Cardiac MRI provides information about the internal structure of CEs and the effects of the cyst on cardiac function.^4^

### Mediastinum

Mediastinum is one of the places where hydatid cyst is very rare. In a study in the literature, mediastinal involvement was reported in approximately 0.5% of thoracic cysts.^75^ In another study, it was emphasized that approximately 0.1% of all cases of hydatid cysts were mediastinal.^76^ Most patients are symptomatic.^77^ Clinical symptoms are cough due to compression, dyspnea, retrosternal or parasternal pain, and dysphagia. Mediastinal echinococcosis is clinically and radiologically indistinguishable from other cystic lesions.^77^ Chest x-ray, CT, and MRI facilitate the diagnosis. Computed tomography is important in showing the morphology, density, and borders of the lesions and revealing their relationship with the surrounding structures. Differential diagnosis can be made by general surgery.^77^

### Pleura

Pleural involvement is usually secondary to lung CE. It is exophytic originating in the liver and towards the chest. Multiple involvement is seen as a result of lung CE rupture.

### Bladder

Primary CE of the bladder is very rare.^58^ Bladder involvement usually occurs secondary to kidney CEs. It is characterized by few clinical symptoms as the cyst remains asymptomatic as a result of slow growth. Hydaturia, which is characterized by the presence of grape skin-like structures in the urine, is the only pathognomonic finding and is evidence of the presence of ruptured hydatid cysts in the urinary tract.^78^

### Ovary

**Figure 14. A,B.**
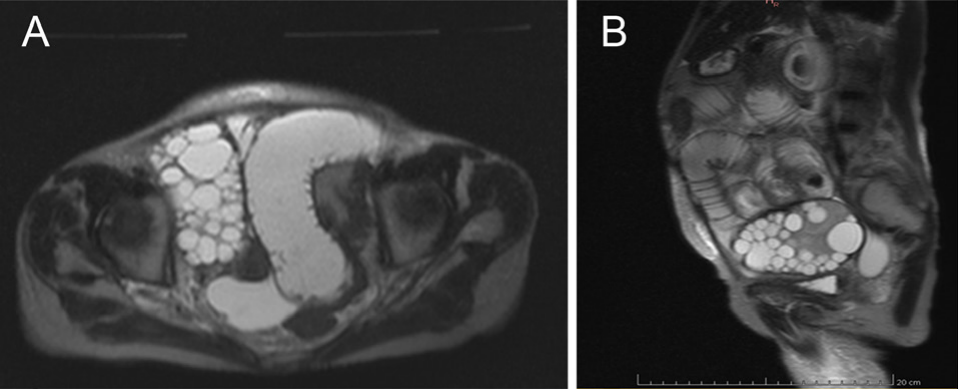
Axial (A) and sagittal (B) T2-weighted magnetic resonance image showing cystic mass with daughter cysts in the right ovary.

Ovary is one of the rarely involved organs in hydatid cyst cases. A few cases of primary involvement of the ovary have been reported in the literature.^79^ Involvement is usually secondary to peritoneal spread. Most cases are recognized during surgery.^80^ Ovarian cyst hydatidosis may not be differentiated from septal ovarian lesions such as cystadenoma and cystic teratoma due to its multilocular cystic structure.^81^ Hydatid cysts grow slowly and symptoms appear after they reach a certain size. Therefore, it can be large at the time of application (Figure 14).

### Scrotum

Cystic echinococcosis of the scrotum is rarely encountered in the literature. Only a few cases have been reported. In most of the cases in the literature, the scrotum is referred to as the secondary site of involvement. A case of primary involvement of the scrotum has also been reported.^82^ It can spread by hematogenous or lymphogenous routes. Imaging findings are similar to those in other organs.

## Conclusion

Cystic echinococcosis is a dynamic entity with different imaging characteristics due to the changing stage of the cyst. After reaching the gastrointestinal tract, it passes into the portal circulation and can be seen anywhere in the body where the blood reaches. Knowing the imaging features and being familiar with the image are very helpful in reaching the diagnosis, especially in endemic areas. Even familiarity with the image may be insufficient to make the diagnosis of cystic lesions encountered in unusual locations. For this reason, CE is one of the important diseases that should be kept in mind when cystic lesions are encountered, especially in endemic areas.

In addition, different treatment approaches are applied to different types of this parasitic cyst. In order to apply the right treatment, after making the diagnosis, making the right decision on the type of cyst with imaging tools is as important as making the diagnosis.

## Figures and Tables

**Table 1.** WHO Classification of Cystic Echinococcosis and Recommended Treatments

Stage	Main Characteristic	Viability	Treatment
Cystic echinococcosis 1	Uniloculated cyst with double wall sign	Active cyst (viable)	ABZ 3-6 months (small cysts < 5 cm) or percutaneous treatment (PA(IR)) and 1-6 months ABZ (cysts 5-10 cm) or surgery + 1-3 month ABZ (cysts > 10 cm)
Cystic echinococcosis 2	Uniloculated cyst with regular, vascular “septations” resembling daughter cysts	Active cyst (viable)	Surgery + 1-3 month ABZ or modified percutaneous treatment + 1-3 month ABZ or ABZ alone 3-6 months if cyst is small
Cystic echinococcosis 3A	Continuous thin and regular membranes floating in the cyst, resembling detached parasite layers	Transitional (variable viability)	ABZ 3-6 months (Small cysts < 5 cm) or percutaneous treatment (PA(IR)) and 1-6 months ABZ (cysts 5-10 cm) or surgery + 1-3 month ABZ (cysts > 10 cm)
Cystic echinococcosis 3B	Predominantly solid with daughter cysts	Transitional (variable viability)	Surgery + 1-3 month ABZ or modified percutaneous treatment + 1-3 month ABZ or ABZ 1-6 months if cyst is small
Cystic echinococcosis 4	Parasitic membranes embedded in heterogeneous, avascular solid content (“ball of wool” appearance)	Inactive (low or no viability)	Watch and wait
Cystic echinococcosis 5	Cysts with solid content with eggshell wall calcifications	Inactive (no viability)	Watch and wait

ABZ, albendazole; PAIR, puncture, aspiration, instillation, and reaspiration; WHO, World Health Organization.

Adapted from Rinaldi et al.^34^

## References

[1] McManus DP Gray DJ Yang Y . Diagnosis, treatment, and management of echinococcosis. BMJ. 2012;344:e3866. 10.1136/bmj.e3866 22689886

[2] Khiari A Mzali R Ouali M Kharrat M Kechaou MS Beyrouti MI . Hydatid cyst of the pancreas. Apropos of 7 cases. Ann Gastroenterol Hepatol (Paris). 1994;30(3):87 91. 8067682

[3] Pedrosa I Saíz A Arrazola J Ferreirós J Pedrosa CS . Hydatid disease: radiologic and pathologic features and complications. Radiographics. 2000;20(3):795 817. (10.1148/radiographics.20.3.g00ma06795) 10835129

[4] Polat P Kantarci M Alper F Suma S Koruyucu MB Okur A . Hydatid disease from head to toe. Radiographics. 2003;23(2):475 94; quiz 536. (10.1148/rg.232025704) 12640161

[5] Tüzün M Hekimoğlu BJ . Pictorial essay. Various locations of cystic and alveolar hydatid disease: CT appearances. J Comput Assist Tomogr. 2001;25(1):81 87. (10.1097/00004728-200101000-00014) 11176298

[6] Kantarci M Onbas O Alper F Celebi Y Yigiter M Okur A . Anaphylaxis due to a rupture of hydatid cyst: imaging findings of a 10-year-old boy. Emerg Radiol. 2003;10(1):49 50. (10.1007/s10140-002-0265-0) 15290532

[7] Schweiger A Ammann RW Candinas D et al. Human alveolar echinococcosis after fox population increase, Switzerland. Emerg Infect Dis. 2007;13(6):878-882. (10.3201/eid1306.061074) PMC2792858 17553227

[8] Drolshammer I Wiesmann E Eckert J . Human echinococcosis in Switzerland during the years 1956-1969. Schweiz Med Wochenschr. 1973;103(40):1386 1392. 4755632

[9] Eckert J et al. WHO/OIE Manual on Echinococcosis in Humans and Animals: a Public Health Problem of Global Concern. World Organisation for Animal Health; 2001.

[10] Kantarci M Pirimoglu B Ogul H et al. Can biliary–cyst communication be predicted by Gd-EOB-DTPA-enhanced MR cholangiography before treatment for hepatic hydatid disease?. Clin Radiol. 2014;69(1):52 58. (10.1016/j.crad.2013.08.005) 24156798

[11] Oral A Yigiter M Yildiz A et al. Diagnosis and management of hydatid liver disease in children: a report of 156 patients with hydatid disease. J Pediatr Surg. 2012;47(3):528 534. (10.1016/j.jpedsurg.2011.11.007) 22424349

[12] Beggs IJ The radiology of hydatid disease. AJR. 1985;145(3):639 648. (10.2214/ajr.145.3.639) 3895873

[13] Tamarozzi F Akhan O Cretu CM et al. Prevalence of abdominal cystic echinococcosis in rural Bulgaria, Romania, and Turkey: a cross-sectional, ultrasound-based, population study from the HERACLES project. Lancet Infect Dis. 2018;18(7):769 778. (10.1016/S1473-3099(18)30221-4) 29793823

[14] Balik AA Celebi F Başglu M Oren D Yildirgan I Atamanalp SS . Intra-abdominal extrahepatic echinococcosis. Surg Today. 2001;31(10):881 884. (10.1007/s005950170027) 11759882

[15] Eren S Yildirgan I Kantarci AM . An asymptomatic ruptured hepatic hydatid cyst case presenting with subdiaphragmatic gas in a traumatic patient. Emerg Radiol. 2005;12(1-2):50 52. (10.1007/s10140-005-0433-0) 16283222

[16] Yildirgan MI Başoğlu M Atamanalp SS et al. Intrabiliary rupture in liver hydatid cysts: results of 20 years’ experience. Acta Chir Belg. 2003;103(6):621 625. (10.1080/00015458.2003.11679505) 14743572

[17] Bedirli A Sakrak O Sozuer EM Kerek M Ince O . Surgical management of spontaneous intrabiliary rupture of hydatid liver cysts. Surg Today. 2002;32(7):594 597. (10.1007/s005950200107) 12111515

[18] WHO Informal Working Group on Echinococcosis. Guidelines for treatment of cystic and alveolar echinococcosis in humans. Bull World Health Organ. 1996;74:231 242.

[19] Chautems R Bühler LH Gold B et al. Surgical management and long-term outcome of complicated liver hydatid cysts caused by *Echinococcus granulosus* . Surgery. 2005;137(3):312 316. (10.1016/j.surg.2004.09.004) 15746785

[20] Ozturk G Aydinli B Yildirgan MI et al. Posttraumatic free intraperitoneal rupture of liver cystic echinococcosis: a case series and review of literature. Am J Surg. 2007;194(3):313 316. (10.1016/j.amjsurg.2006.11.014) 17693274

[21] Bristow BN et al. Human echinococcosis mortality in the United States, 1990–2007. PLoS Negl Trop Dis. 2012;6(2):e1524. 10.1371/journal.pntd.0001524 PMC3274497 22347516

[22] Brunetti E Cystic echinococcosis. Tropical Diseases in Travelers. 2009:264 274.

[23] Brunetti E Kern P Vuitton DA Writing Panel for the WHO-IWGE. Expert consensus for the diagnosis and treatment of cystic and alveolar echinococcosis in humans. Acta Trop. 2010;114(1):1 16. (10.1016/j.actatropica.2009.11.001) 19931502

[24] Agudelo Higuita NI Brunetti E McCloskey CJ . Cystic echinococcosis. J Clin Microbiol. 2016;54(3):518 523. (10.1128/JCM.02420-15) 26677245 PMC4767951

[25] Mariconti M Bazzocchi C Tamarozzi F et al. Immunoblotting with human native antigen shows stage-related sensitivity in the serodiagnosis of hepatic cystic echinococcosis. Am J Trop Med Hyg. 2014;90(1):75-79. (10.4269/ajtmh.13-0341) PMC3886432 24297816

[26] Peters L Burkert S Grüner BJ . Parasites of the liver – epidemiology, diagnosis and clinical management in the European context. J Hepatol. 2021;75(1):202 218. 10.1016/j.jhep.2021.02.015 33636243

[27] El Fortia M El Gatit A Bendaoud M . Ultrasound wall-sign in pulmonary echinococcosis (new application). 2006;27(06):553 557. 10.1055/s-2006-927232 17160760

[28] Stojkovic M Junghanss T Veeser M Weber TF Sauer P . Endoscopic treatment of biliary stenosis in patients with alveolar echinococcosis – report of 7 consecutive patients with serial ERC approach. PLoS Negl Trop Dis. 2016;10(2):e0004278. (10.1371/journal.pntd.0004278) PMC4766234 26910822

[29] Hosch W Junghanss T Stojkovic M et al. Metabolic viability assessment of cystic echinococcosis using high-field 1H MRS of cyst contents. NMR Biomed. 2008;21(7):734 754. (10.1002/nbm.1252) 18384178

[30] Hosch W Stojkovic M Jänisch T Kauffmann GW Junghanss T . The role of calcification for staging cystic echinococcosis (CE). Eur Radiol. 2007;17(10):2538 2545. (10.1007/s00330-007-0638-6) 17473925

[31] Çankaya BY Yeşilyurt M Giant hydatid cysts in pregnancy: a rare presentation. Rev Soc Bras Med Trop. 2021; 54;e0500-2020 . 10.1590/0037-8682-0500-2020 PMC8008922 33681921

[32] de Diego Choliz J Lecumberri Olaverri FJ Franquet Casas T Ostiz Zubieta S . Computed tomography in hepatic echinococcosis. AJR. 1982;139(4):699 702. (10.2214/ajr.139.4.699) 6981931

[33] Ran B Aji T Jiang T et al. Differentiation between hepatic cystic echinococcosis types 1 and simple hepatic cysts: a retrospective analysis. Medicine (Baltimore). 2019;98(1):e13731. (10.1097/MD.0000000000013731) 30608385 PMC6344192

[34] Rinaldi F Brunetti E Neumayr A Maestri M Goblirsch S Tamarozzi F . Cystic echinococcosis of the liver: a primer for hepatologists. World J Hepatol. 2014;6(5):293-305. (10.4254/wjh.v6.i5.293) PMC4033287 24868323

[35] WHO Informal Working Group. International classification of ultrasound images in cystic echinococcosis for application in clinical and field epidemiological settings. Acta Trop. 2003;85(2):253 261. 12606104 10.1016/s0001-706x(02)00223-1

[36] Kabaalioğlu A Ceken K Alimoglu E Apaydin A . Percutaneous imaging-guided treatment of hydatid liver cysts: do long-term results make it a first choice?. Eur J Radiol. 2006;59(1):65 73. (10.1016/j.ejrad.2006.01.014) 16513311

[37] Koken D Cagli B Tuncel SA Sengul E Yilmaz E Unlu ME . Efficacy of diffusion-weighted MRI in the differentiation of all liver hydatid cyst types. J Med Imaging Radiat Oncol. 2016;60(1):59 65. (10.1111/1754-9485.12417) 26597563

[38] Gharbi HA Hassine W Brauner MW Dupuch K . Ultrasound examination of the hydatic liver. Radiology. 1981;139(2):459 463. (10.1148/radiology.139.2.7220891) 7220891

[39] Bezzi M Teggi A De Rosa F et al. Abdominal hydatid disease: US findings during medical treatment. Radiology. 1987;162(1 Pt):91 95. (10.1148/radiology.162.1.3538157) 3538157

[40] Lee JK Computed Body Tomography with MRI Correlation. Philadelphia: Lippincott Williams & Wilkins; 2006;1.

[41] Frider B Losada CA Larrieu E de Zavaleta O . Asymptomatic abdominal hydatidosis detected by ultrasonography. Acta Radiol. 1988;29(4):431 434. 3044410

[42] Frider B Larrieu E Odriozola MJJoh . Long-term outcome of asymptomatic liver hydatidosis. J Hepatol. 1999;30(2):228 231. (10.1016/s0168-8278(99)80066-x) 10068100

[43] Wen H Vuitton L Tuxun T et al. Echinococcosis: advances in the 21st century. Clin Microbiol Rev. 2019;32(2):e00075-18. (10.1128/CMR.00075-18) PMC6431127 30760475

[44] Kurt N Oncel M Gulmez S Ozkan Z Uzun H . Spontaneous and traumatic intra-peritoneal perforations of hepatic hydatid cysts: a case series. J Gastrointest Surg. 2003;7(5):635 641. (10.1016/s1091-255x(02)00434-1) 12850676

[45] Budke CM Carabin H Ndimubanzi PC . A systematic review of the literature on cystic echinococcosis frequency worldwide and its associated clinical manifestations. Am J Trop Med Hyg. 2013;88(6):1011 1027. 10.4269/ajtmh.12-0692 23546806 PMC3752796

[46] Kilani T El Hammami S Horchani H et al. Hydatid disease of the liver with thoracic involvement. World J Surg. 2001;25(1):40 45. 10.1007/s002680020006 11213155

[47] Ramos G Orduña A García-yuste MJWjosM . Hydatid cyst of the lung: diagnosis and treatment. World J Surg. 2001;25(1):46-57. (10.1007/s002680020007) 11213156

[48] Rawat S Kumar R Raja J Singh RS Thingnam SKS . Pulmonary hydatid cyst: review of literature. J Family Med Prim Care. 2019;8(9):2774-2778. (10.4103/jfmpc.jfmpc_624_19) PMC6820383 31681642

[49] Aydinli B Ozturk G Polat KY et al. Extravisceral primary hydatid cyst of the retroperitoneum. ANZ J Surg. 2007;77(6):455 459. (10.1111/j.1445-2197.2007.04094.x) 17501886

[50] Kosmidis C Efthimiadis C Anthimidis G et al. Management of peritoneal hydatid cysts: a forty-year experience. Heliyon. 2018;4(12):e00994. 10.1016/j.heliyon.2018.e00994 PMC6280071 30555954

[51] Karavias DD Vagianos CE Kakkos SK Panagopoulos CM Androulakis JA . Peritoneal echinococcosis. World J Surg. 1996;20(3):337 340. (10.1007/s002689900054) 8661841

[52] Izgi E Ogul H Aydin Y . Giant peritoneal hydatid cyst causing pelvic venous congestion. Rev Soc Bras Med Trop. 2022;55:PMC9592106. 10.1590/0037-8682-0349-2022 PMC9592106 36287477

[53] Arikanoglu Z Taskesen F Gumus H et al. Selecting a surgical modality to treat a splenic hydatid cyst: total splenectomy or spleen-saving surgery? J Gastrointest Surg. 2012;16(6):1189 1193. (10.1007/s11605-012-1837-2) 22350726

[54] Franquet T Montes M Lecumberri FJ Esparza J Bescos JM . Hydatid disease of the spleen: imaging findings in nine patients. AJR. 1990;154(3):525 528. (10.2214/ajr.154.3.2106214) 2106214

[55] Ödev K Kilinc M Arslan A et al. Renal hydatid cysts and the evaluation of their radiologic images. Eur Urol. 1996;30(1):40 49. (10.1159/000474143) 8854066

[56] Göğüş C Safak M Baltaci S Türkölmez K . Isolated renal hydatidosis: experience with 20 cases. J Urol. 2003;169(1):186 189. (10.1016/s0022-5347(05)64064-5) 12478132

[57] Adanur S Koç E Ziypak T Yapanoglu T Polat O . Giant isolated renal cyst hydatid: from diagnosis to treatment. Arch Ital Urol Androl. 2014;86(2):144 145. (10.4081/aiua.2014.2.144) 25017600

[58] Hertz M Zissin R Dresnik Z Morag B Itzchak Y Jonas P . Echinococcus of the urinary tract: radiologic findings. Urol Radiol. 1984;6(3-4):175 181. (10.1007/BF02923719) 6393527

[59] Engin G Acunaş B Rozanes I Acunaş G . Hydatid disease with unusual localization. Eur Radiol. 2000;10(12):1904 1912. (10.1007/s003300000468) 11305568

[60] Torres AR Microbiología y Parasitología Médica. Salvat; 1995.

[61] Torricelli P Martinelli C Biagini R Ruggieri P De Cristofaro R . Radiographic and computed tomographic findings in hydatid disease of bone. Skeletal Radiol. 1990;19(6):435 439. (10.1007/BF00241799) 2218593

[62] Kalovidouris A Pissiotis C Pontifex G Gouliamos A Pentea S Papavassiliou C . CT characterization of multivesicular hydatid cysts. J Comput Assist Tomogr. 1986;10(3):428 431. 3700744

[63] Işlekel S Erşahin Y Zileli M et al. Spinal hydatid disease. Spinal Cord. 1998;36(3):166 170. (10.1038/sj.sc.3100512) 9554015

[64] Ismail K Haluk GI Necati OJIs . Surgical treatment of hydatid cysts of the pancreas. Int Surg. 1991;76(3):185 188. 1938210

[65] Safioleas MC Moulakakis KG Manti C Kostakis A . Clinical considerations of primary hydatid disease of the pancreas. Pancreatology. 2005;5(4-5):457 461. (10.1159/000086548) 15985772

[66] Schoretsanitis G de Bree E Melissas J Tsiftsis D . Primary hydatid cyst of the adrenal gland. Scand J Urol Nephrol. 1998;32(1):51 53. (10.1080/003655998750014693) 9561575

[67] Bastounis E Pikoulis E Leppániemi A Cyrochristos D . Hydatid disease: a rare cause of adrenal cyst. Am Surg. 1996;62(5):383 385. 8615568

[68] Yeniyol CO Minareci S Ayder AR . Primary cyst hydatid of adrenal: a case report. Int Urol Nephrol. 2000;32(2):227 229. (10.1023/a:1007106328997) 11229636

[69] Onbas O Kantarci M Alper F Sekmenli N Okur A . Spinal widespread intradural extramedullary hydatidosis. Neuroradiology. 2004;46(4):310 312. (10.1007/s00234-004-1184-4) 15045497

[70] Yörükoglu Y Zengin M Dolgun A et al. Primary muscular hydatid cyst causing arterial insufficiency: case report and literature review. Angiology. 1993;44(5):399 401. (10.1177/000331979304400509) 8480918

[71] Bayram M Sirikci AJEjor . Hydatic cyst located intermuscular area of the forearm: MR imaging findings. Eur J Radiol. 2000;36(3):130 132. (10.1016/s0720-048x(00)00188-1) 11091011

[72] Dasbaksi K Haldar S Mukherjee K Mukherjee P . A rare combination of hepatic and pericardial hydatid cyst and review of literature. Int J Surg Case Rep. 2015;10:52 55. (10.1016/j.ijscr.2015.02.052) 25805610 PMC4429948

[73] Yilmaz E Osma E Balci PJER . Cardiopulmonary echinococcosis: MR assessment. Eur Radiol. 2000;10(9):1504. (10.1007/s003300000339) 10997447

[74] Yekeler I Koçak H Aydin NE et al. A case of cardiac hydatid cyst localized in the lungs bilaterally and on anterior wall of right ventricle. Thorac Cardiovasc Surg. 1993;41(4):261 263. (10.1055/s-2007-1013868) 8211934

[75] Thameur H Chenik S Abdelmoulah S et al. Thoracic hydatidosis. A review of 1619 cases. Rev Pneumol Clin. 2000;56(1):7 15. 10740109

[76] Rakower J HJTAJoM . Milwidsky. Prim Mediastinal Echinococcosis. 1960;29(1):73 83. 10.1016/0002-9343(60)90008-5 14435973

[77] Eroğlu A Kürkçüoğlu C Karaoğlanoğlu N Tekinbaş C Kaynar H Onbaş O . Primary hydatid cysts of the mediastinum. Eur J Cardiothorac Surg. 2002;22(4):599 601. (10.1016/s1010-7940(02)00398-6) 12297179

[78] Sallami S Nouira Y Kallel Y Gargouri M Horchani A . Intravesical hydatid cyst. Urology. 2005;66(5):1110. (10.1016/j.urology.2005.06.072) 16286147

[79] Hangval H Habibi H Moshref A Rahimi A . Case report of an ovarian hydatid cyst. J Trop Med Hyg. 1979;82(2):34 35. 458904

[80] Ray S Gangopadhyay M . Hydatid cyst of ovary-a rare entity. J Turk Ger Gynecol Assoc. 2010;11(1):63. PMC3939309 24591898

[81] Vashist M Godara R . Ovarian carcinoma mimicking peritoneal hydatidosis: an unusual case. Internet J Gynecol Obstet. 2007;6(2).

[82] Bouchikhi AA Lamrani YA Tazi MF et al. Unilateral primitive hydatid cyst with surgical resection of the scrotum: a case report. J Med Case Rep. 2013;7(1):109. (10.1186/1752-1947-7-109) 23601913 PMC3639827

